# The role of PPAR in fungal keratitis

**DOI:** 10.3389/fimmu.2024.1454463

**Published:** 2024-12-23

**Authors:** Hongyan Zhou, Hong Zhang, Miaomiao Bi, Wensong Zhang

**Affiliations:** ^1^ Department of Ophthalmology, The China-Japan Union Hospital of Jilin University, Changchun, Jilin, China; ^2^ Department of Ophthalmology, The Second Hospital of Jilin University, Changchun, Jilin, China

**Keywords:** PPAR, fungal keratitis, infection and inflammation, cytokines, oxidative stress, immunology

## Abstract

The treatment of fungal keratitis(FK) remains challenging due to delayed fungal detection and the limited effectiveness of antifungal drugs. Fungal infection can activate both innate and adaptive immune responses in the cornea. Fungi stimulate the production of oxidative stress-related biomarkers and mediate the infiltration of neutrophils, macrophages, and T cells. These cells can induce infiltration of cytokines, chemokines, and matrix metalloproteinases (MMPs), leading to corneal tissue damage and even corneal perforation. The signaling pathway regulates the expression of inflammatory cytokines in fungal keratitis. Immune inflammatory damage is the main mechanism of FK, and oxidative stress damage is also involved in this infection process. Peroxisome proliferator-activated receptor (PPAR) is a member of the nuclear hormone receptor superfamily, with different subtypes of PPAR a, PPAR β/δ, and PPARγ. PPARs play important roles in the antioxidant response, anti-inflammatory, lipid metabolism, neuroprotection, and immune regulation processes. PPAR γ can promote macrophage polarization and reduce oxidative stress damage by regulating ROS production. PPAR has made some progress in the treatment of eye diseases: PPARa agonists can inhibit diabetes keratopathy and corneal neuropathy. PPARa agonists inhibit early immature angiogenesis in corneal alkali burns and have potential therapeutic effects on inflammatory corneal angiogenesis. PPARs can control the progression of dry eye disease and improve the condition of meibomian gland dysfunction. Based on this, we explored the potential roles of PPARs in the treatment of FK.

## 1 Introduction

The treatment of fungal infections is an important part of global public health. Fungi is the most important environmental biomarker. Its biodiversity is very important and highly sensitive to environmental changes. Fungal resistance can lead to uncontrolled infections that can be life-threatening (1, 2). Microbial keratitis includes bacteria, viruses, fungi, and protozoa. The incidence of FK is increasing year by year. FK accounts for 40% to 50% of all cases of microbial keratitis. FK can cause corneal damage and endophthalmitis, which can eventually lead to vision loss. The main causative agents of FK are Fusarium and Aspergillus (3, 4). Trauma, weakened immune function, ocular surface disease, and wearing contact lenses are the main causes of FK. The lag of fungal test results is challenging (5). Fungal resistance and corneal transplant rejection are currently challenges in the treatment of aggressive FK (6).The difficulties of drug therapy with FK lie in the limited permeability of antifungal drugs, low bioavailability, poor pharmacokinetic properties and corneal toxicity. The lack of donors for corneal transplantation is a dilemma for fungal treatment (7).

Exploring new adjuvant treatment options in clinical practice is a top priority. PPAR can be a good option. PPARs are the members of nuclear hormone receptor superfamilies with different subtypes PPAR α, PPAR β/δ, and PPAR γ. PPARs can regulate energy homeostasis, and oxidative stress, and determine cell fate (8). Impaired peroxidase function can lead to different diseases, which are associated with severe clinical symptoms. Oxidative stress damage is also involved in the pathogenesis of FK (9). Pro-inflammatory cytokines, oxidative stress damage, macrophage reactive oxygen species, and mitochondrial reactive oxygen species levels during FK pathogenesis are our therapeutic targets (10). PPAR has definite anti-inflammatory and antioxidant effects. One of the key functions of peroxisomes is the oxidation of fatty acids, which can be mobilized and transferred to peroxisomes and mitochondria, thus efficiently exchanging metabolites (11, 12). Excessive inflammation and immune damage during FK can cause irreversible damage to corneal tissue. PPARa is a transcription factor that can modulate inflammation. PPARα functional modulators inhibit inflammation by blocking peripheral immune cells and the Toll-like receptor-4(TLR-4)/the nuclear factor-κB(NF-κB)pathway (13). Numerous studies suggest that PPARγ agonists may be effective therapeutic agents for inflammation-related diseases (14). PPAR activators inhibit the expression of inflammatory response genes such as IL-2, IL-6, IL-8, TNFα, and MMP by inhibiting the NF-κB signal transducer, the activator of the transcription(STAT) and Activator Protein -1(AP-1) signaling pathways (15).

Finding appropriate reagents to fight inflammation, modulate an overactive immune response, and resist oxidative stress damage is essential to prevent infection-related corneal damage. Peroxisome could be the ideal target. In this review, we emphasize corneal fungal infections that require progress to better express treatment of FK related to PPARs.

## 2 Immunity to fungi in the cornea

Fungi infection activated the corneal innate and adaptive immune response (16).

### 2.1 Innate immunity to FK

Innate immunity is the host’s first line of defense against microbial infection. Fungal infections in immunocompromised patients can cause severe tissue damage. The corneal cells specifically recognizes patterns associated with molecular patterns (PAMPs) on the fungal surface through pattern recognition receivers (PRRs). PRRs play a clinical role in innate immunity, mediating the infiltration of neutrophils, macrophages, and T cells, and ultimately the destruction of fungi. The infiltration of these cells can lead to infiltration of cascades of amplified inflammatory and chemokines, which can eventually lead to corneal tissue damage and even corneal perforation. The innate immune function relies on the recognition of PAMPs, and damage-associated molecular patterns (DAMPs) of pathogen by PRRs (17, 18). Ficolin-A (FCN-A) is a class of soluble PRRs that play an important role in innate immunity. Downregulation of FCN-A reduces the inflammatory response and decreases the expression of tumor necrosis factor(TNF-a), p-p38, p-JNK, and mitogen-activated protein kinase(MAPK)in corneal infected mice, and promotes macrophage polarization (19). The proportion of dendritic cells(DCs) in the early stage of FK generally decreases with fungal infection, while the proportion of macrophages, monocytes, and neutrophils increases dramatically in the early stage and then gradually decreases as inflammation resolves. Activation of adaptive immune cells is also observed in the later stages of infection. Activation of early acquired immune cells favors fungal clearance, which gradually decreases in the later stages of inflammation to reduce the damage to corneal tissue by the corresponding cytokines (20). β-glucan on A. fumigatus germinate conidia which activates Dectin-1 on corneal macrophages produce interleukin-1(IL-1β), and CXC ligand-1(CXCL_1_), recruiting neutrophils from the corneal stroma through IL-1R_1_/myeloid differentiation primary response gene 88 (MYD88) dependent activation and killing fungi through the TLR4 dependent pathway (21). Fumigatus-infected corneal DCs could drive naive CD4+ T-cell proliferation and promote the production of Th17 cytokines and IL-22. Activated DC produces thymic stromal lymphopoietin (TSLP) leading to a Th17-type inflammatory response via the Janus kinase-signal transducer and activator of transcription pathway(JAK/STAT)signaling pathway (22). Fumigatus-infected mouse cornea induces macrophage infiltration through a TLR-4-mediated signaling pathway. and produce pro-inflammatory factors IL-1β, tumour necrosis factor-α(TNF-α), and IL-6 in the corneal epithelium and stroma. The antifungal and anti-inflammatory reagents should be found in the therapy of FK (23).T cells follicular helper, monocytes, macrophages, and mast cells might play a key role in Fusarium keratitis using analysis of immune infiltration (24). Inhibition of macrophage phagocytosis and killing function can mediate the protective immune effect of Aspergillus fumigatus (25, 26). In the process of FK infection, finding effective macrophage pathogen clearance while reducing unnecessary inflammatory reactions to alleviate tissue damage is the immune regulatory target that scholars are dedicated to searching for (27).

### 2.2 Adaptive immunity to FK

#### 2.2.1 Cellular immunity

Immunity through the mucosal surface stimulates IgA antibodies in tears which can prevent the development of keratitis (28). T helper cells(Th17)and regulatory T (Treg)cells share a common precursor cell (naive CD4 T cells), Th17 originated from neutrophils, plays a key role in the severity of autoimmune and inflammatory infection, while Treg cells inhibit Immune inflammatory damage and maintain immune homeostasis.Th17/Treg cells balance signaling pathways were triggered by T cell receptors, costimulatory receptors, and cytokines, as well as various metabolic pathways and gut microbiota (29). CD4+T cell-driven inflammation leads to irreversible damage to the cornea. and can lead to corneal nerve damage (30, 31). The regulation of Th17/Treg differentiation will determine the outcome of fungal keratitis. This will be a potential site (32, 33).

#### 2.2.2 Humoral immunity

Local and systemic immunity inhibits pathogen responses in some keratitis, such as infectious bovine keratoconjunctivitis (34). Anti-amoebic antigens were detected in tear and ocular lysates, and different levels of Immunoglobulin M(IGM) and Immunoglobulin A(IGA) antibodies were detected during the course of the disease (35). The keratitis vaccine has not been clinically promoted. Treatment of corneas infected with Pseudomonas aeruginosa was effective with immune serum from animals immunized with Pseudomonas aeruginosa membrane vesicles, and the results were effective. Topical treatment with immune serum reduces corneal clinical scores (36).

## 3 Corneal inflammations in FK

The treatment of FK needs to be based on preventing spore adhesion and disrupting the formation of fungal biofilms, reducing the expression of inflammatory factors in corneal epithelial cells, and inducing reactive oxygen species (ROS) levels in corneal epithelial cells (24). Many cytokines and chemokines are involved in the progression of FK. NF-κB pathway, phosphatidylinositol-3 kinase (PI3K)/protein kinase B(AKT) pathway like PI3K and AKT, and MAPK pathway like p38 regulate the expression of inflammatory cytokines. The fungus induces an increase in TLR4/MyD88 expression in corneal tissue. It can be used as an effective way of action against fungal infection (37, 38). In infectious keratitis, corneal stromal MMPs, especially MMP-9 and MMP-2, increase activity, inducing corneal collagen degradation and ulcer formation (39). Macrophage inhibitory protein 2 (MIP-2) and intercellular adhesion molecule 1 (ICAM-1) attract neutrophils to the inflammatory site in the cornea (40, 41). Neutrophils are the earliest cells in the body’s immune response and are an important part of fighting pathogens (42). The corneal inflammatory response to fungal infection is caused by the accumulation of inflammatory cells. Neutrophils trigger the expression of cytokines and chemokines. These cytokines and chemokines deposit immune cells, and excessive immune responses can cause immune damage, corneal opacity, and vision loss. Therefore, anti-inflammatory is an important direction of FK treatment (43–46). Downregulated the p-p65/p65 and p-IκB/IκB protein ratios, attenuate the inflammatory response and fungal burden in FK (47). DC-derived TSLP promotes the Th17-type inflammatory response through the JAK/STAT signaling pathway. Corneal damage in Aspergillus fumigatus-infected keratitis can be reversed by inhibition of the JAK/STAT signaling pathway with specific inhibitors (22).

## 4 Oxidative stress damage in FK

**Figure 1:**
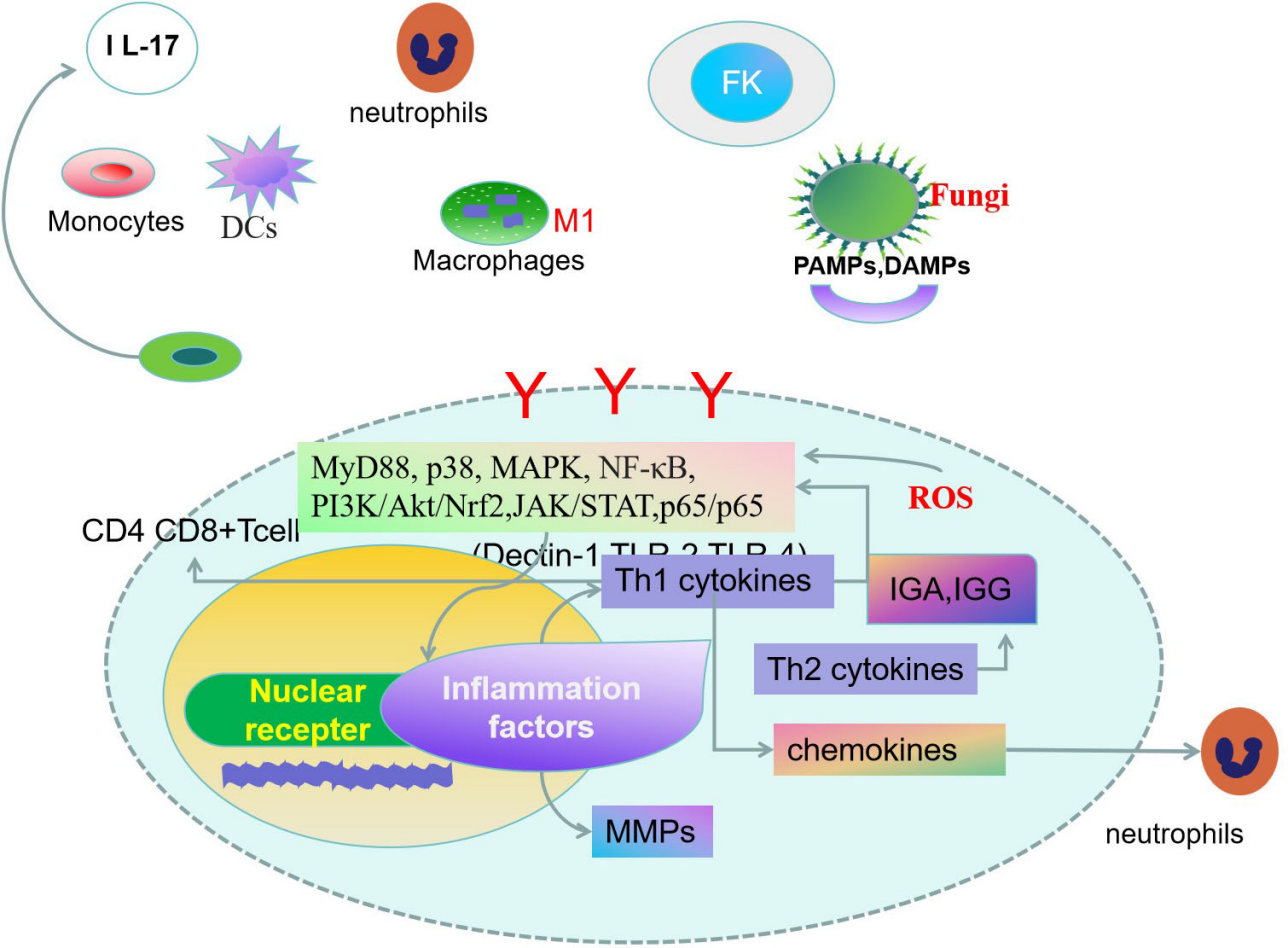
Innate and adaptive immune response are both involved in the pathogenesis of fungal keratitis(FK) (16). Corneal cells specifically recognize fungal-associated patterns associated with molecular patterns (PAMPs), damage-associated molecular patterns (DAMPs) molecules by pattern recognition receivers (PRRs) (17, 18). Dendritic cells(DCs) can drive naïve CD4+ T cell proliferation, leading to inflammatory responses by activating signaling pathways. T helper cells, monocytes, macrophages, and mast cells play key roles in FK. Activation of signal transduction molecules promotes the expression of cytokines, chemokines, and rmatrix metalloproteinases (MMPs) (22, 24). The fungus stimulates the production of oxidative stress-related markers in corneal cells. Inhibition of oxidative stress damage will be an effective way to alleviate corneal damage in fungal infections (52).

The fungus stimulates the production of oxidative stress-related markers (ROS, HNE, NO, MDA mitochondrial DNA 8-OHdG, and aconitase-2) in corneal cells. Fungal infections also increase HMOX1 and COX2 expressions and inhibit the levels of antioxidant enzymes, superoxide dismutase-1 (SOD1), glutathione peroxidase-1 (GPx1), and peroxide resin-4 (PRDX4) (48). Upregulation of HO-1 leads to the conversion of heme to carbon monoxide, which acts as an inhibitor of the NF-κB pathway, leading to decreased expression of pro-inflammatory cytokines. Inflammation is the main culprit in exacerbating fungal infections (49, 50). Activation of the Nrf2/(HO)-1 pathway reduces the innate immune response mediated by oxidative stress.Nrf2 plays an important role in promoting corneal wound healing (51). Inhibition of oxidative stress damage is an effective way to alleviate corneal damage in fungal infections (52) (**Figure 1**).

## 5 The potential role of PPARs in FK

The anti-inflammatory and antioxidant effects of PPAR agonists in various inflammatory diseases have been confirmed, but their roles in FK is worth exploring.

### 5.1 The role of peroxisome in liver disease

PPAR enhances the production of high-density lipids and inhibits the proliferation of vascular smooth muscle cells. PPARα prevents cardiac complications through adenosine-activated protein kinase, TGF-β activated kinase, and NF-κB pathway (53). PPARα/γ agonists can reduce cardiovascular risk by improving the serum atherosclerotic lipoprotein profile of patients with nonalcoholic fatty liver disease (54). The dual agonist of PPAR a,δ, efebranol, can significantly improve the relevant biochemical indicators of cholestasis and is expected to treat primary biliary cholangitis caused by liver fibrosis (55, 56).

### 5.2 The role of peroxisome in nerve protection

PPARγ agonists are being studied for the treatment of neurodegenerative diseases including Alzheimer’s and Parkinson’s disease (57, 58). The biology of PPARγ inhibits transmembrane spanning transporters or alpha-amino-3-hydroxy-5-methyl-4-isoxazolepropionic acid(AMPA) receptors thus alleviating brain swelling and changes around tumors (57–59). PPARγ coactivator 1α plays an important role in maintaining mitochondrial function, protecting peripheral nerves, and alleviating chemotherapy-induced peripheral neuropathy (60). PPARa is selectively expressed in certain brain regions and neuronal glial cells and regulates antioxidant responses, neurotransmission, neuroinflammation, neurogenesis, and glial cell proliferation differentiation. PPAR agonists perform a protective role in neurodegenerative diseases and neuropsychiatric disorders (61).

### 5.3 The role of peroxisome in inflammatory diseases

PPAR is a ligand-dependent transcription factor belonging to the nuclear receptor family. The basic function of PPARα is to regulate the oxidation of fatty acids. PPARβ/δ is involved in lipid metabolism, wound healing, embryonic development, and inflammation (62–64). PPAR agonists have recently demonstrated promising short-term biochemical responses in patients with primary cholangitis (65). PPARγ performs anti-inflammatory and anti-fibrotic effects in different disease models (64). PPARγ agonists can be used to alleviate allergic inflammation and inhibit pro-inflammatory gene expression programs (66). Recombinant Human Fibroblast Growth Factor 21(rhFGF21)Regulates the secretion of pro- and anti-inflammatory cytokines to improve neurological deficits in behavioral testing. It attenuates macrophage polarization to the M1 phenotype and peripheral immune cell accumulation, inhibits NF-κB, and upregulates PPARγ to inhibit pro-inflammatory cytokine expression (67). In the treatment of immunoglobulin A nephropathy rat model, the addition of Yi-Shen-Hua-Shi granules modulates immune and inflammatory damage by the PPARγ/NF-κB pathway (68). PPAR-γ is a transcription factor with anti-inflammatory capacity. Nuclear receptors are important bridges between lipid signaling molecules and transcription responses. Macrophages downregulate PPARγ and its target genes under inflammatory conditions, thereby decreasing lipid stress in these cells. PPARγ is required during the resolution phase of the inflammatory response, and the absence of PPARγ is associated with a sustained immune response. Signaling pathways (p38, NFκB, ERK1/2) activated by TLR_2_ and TL_4_ were inhibited by PPARγ in various situations (69–72).

### 5.4 Existing research results of peroxisome in eye diseases

Both PPARα and PPARγ could be detected in the cornea, conjunctiva, meibomian gland, and lacrimal gland. PPARγ is expressed at higher levels than PPARα in all tissues (73).

#### 5.4.1 Dry eye

PPARα, β/δ, γ can be expressed in the conjunctiva and lacrimal glands. Decreased tear secretion, shortened BUT, and increased corneal staining were shown in Dry eye rats and diabetic rats. The expression of PPARγ decreased, while the expression of TNF-α, IL-1β, IL-6, MyD88, and TGF-β increased in both dry-eye rats and diabetic rats (74). Excessive keratinization of the meibomian gland epithelium leads to obstruction of the meibomian opening, meibomian stasis, and cystic dilation of the ducts, resulting in secondary disuse acinar atrophy and glandular shedding (75). PPARγ is responsible for the formation of ductal lumens and the differentiation of epithelial cells into meibocyte phenotype during meibomian gland development. The decrease in PPARγ receptor expression is the key role of meibomian gland atrophy and degeneration (76). PPAR ligands have neuroprotective and anti-inflammatory effects (77), making them a promising drug choice for the treatment of dry eye syndrome.

#### 5.4.2 Corneal alkali burn

In the rat corneal alkali burn model, PPARα, PPARγ agonists, and their combinations of ophthalmic fluid were administered respectively in the corneal alkali burn model. PPARα, PPARγ, and PPARα,γ agonists inhibit inflammatory cells, neovascularization, and fibrotic changes. PPARγ agonists facilitate M_2_ macrophages and promote wound healing, The combination of PPARα with γ agonists may be a new therapy strategy for corneal alkali burns (78, 79). The increased expression level of PPARγ mRNA can serve as one of the factors for evaluating the prognosis of corneal alkali burn (80).

PPARγ can reduce the expression of downstream inflammatory molecules by regulating signaling molecules. The enhanced expression of PPARγ protein in spinal microglia can inhibit the activity of the NF - κ B pathway, increase the expression of M2 polarization-related genes and significantly reduce the level of inflammatory factors (81). Oxidative stress and inflammatory reaction play an important role in the development of diabetes. Curcumin has been shown to have anti-inflammatory effects in combating changes induced by hyperglycemia by regulating signaling pathways such as PPAR - γ and NF - κ B, as well as high expression of IL-1 β, IL-6, and chemokines such as MCP-1 (82). By regulating the polarization of M2 macrophages through the PPARγ/NFκB axis and IL-6/IL-6R signaling pathway, the proliferation, migration, and glucose metabolism of lung adenocarcinoma cells are regulated (83).

#### 5.4.3 corneal epithelial injury

In a model of delayed corneal epithelial injury, PPAR agonists or vehicle ophthalmic fluids are locally instilled into the rat cornea, and the corneal epithelial healing process is assessed by fluorescein staining. The area of corneal epithelial defect in the PPAR agonist ophthalmic fluid group was significantly smaller than that in the vehicle group. PPARγ administration can inhibit the expression of inflammatory cytokines and NF-κB thus promoting corneal epithelial healing (84). The expression of PPARγ and PPARβ/δ is reduced in acute epidermal barrier injury, promoting the progression of the disease (85). PPARβ/δ agonists promote the infiltration of M_2_ macrophages and vascular endothelial cells and promote the expression of vascular endothelial growth factor A mRNA. Thus, PPARβ/δ ligands promote corneal neovascularization as well as wound healing processes (86).

#### 5.4.4 Diabetic nerve damage

The strong nerve innervation of the cornea ensures its maintenance of the ocular surface homeostasis. Corneal diabetes neuropathy changes the shape and density of the intranasal plexus and nerves. The length and the fiber density of corneal nerve fiber, corneal nerve branch density, twist coefficient, and bead frequency are quantitative analysis parameters of nerve fibers in corneal diabetes neuropathy (87). PPARα agonists stimulate corneal nerve regeneration, reduce nerve edema, significantly improve corneal nerve fiber density and corneal nerve fiber width, and upregulate the neurotrophic signaling pathway (88). The protective effect of PPARα on diabetes keratopathy and corneal neuropathy is achieved by restoring the level of neurotrophic factors in the cornea. The density of corneal nerve fibers in PPARα -/- mice gradually decreased. The corneal sensitivity of PPARα -/- mice continues to decrease. PPARα agonist has therapeutic potential in the treatment of diabetes keratopathy (89). Fenofibrate(PPAR alpha agonist )can delay the development of retinopathy and reduce macular edema, reducing the demand for laser therapy (90, 91). Fenofibrate has good effects on lipid control, inflammation, angiogenesis, and cell apoptosis. These factors are considered important in the development of DR. Fenofibrate has the function of protecting the retina (92). Fenofibrate is the treatment choice for DR. More understanding of biomarkers related to inflammation and angiogenesis in diabetes retinopathy will help to develop treatment methods for advanced retinopathy (93).

### 5.5 The possible mechanism of PPARs involving in FK

PPARs play multiple roles in the β - oxidation of fatty acids and the metabolism of arachidonic acid metabolites. PPAR is also a key regulatory factor in controlling inflammation and has great therapeutic potential in inflammatory diseases (14). Given this, based on our analysis of the aforementioned literature, we attempt to demonstrate the therapeutic potential of PPAR in FK.

#### 5.5.1 Immune regulation of PPARs

Immune damage plays a crucial role in the pathogenesis of FK. In the early stage of inflammation, the innate immune response plays a protective role, while in the late stage of inflammation, immune damage leads to the formation of corneal ulcers and severe decline in vision. PPARγ regulates macrophage polarization, DC function, and T-cell activation (64). PPARα is an important target through which Melatonin regulates autophagy to alleviate the hepatocyte lipid accumulation of Cadmium (94).

PPARα is an important player in innate immune regulation. To distinguish between self and foreign molecules and cells, the pattern recognition receptor(PRR) binds specific molecule PAMPs from certain groups of common pathogens of viral, bacterial, or fungal origin. In the event of an invasion, phagocytosis is triggered and the production of cytotoxic compounds helps to destroy the engulfed particles. PPARα acts as a transcription factor to exert a strong influence on intracellular signal transduction events, fighting a disruptive cytokine storm (95, 96). Peroxisomes are key regulators that regulate immunity, paving the way for potential therapeutic intervention in FK by its immunomodulatory function.

#### 5.5.2 Inhibition effect of PPARs on oxidative stress damage

Oxidative stress promotes the formation of liver fibrosis. Inhibition of ROS-mediated effects and modulation of major antioxidant responses has emerged as therapeutic targets for the prevention of liver injury. ROS is involved in signal transduction, which regulates inflammation and immune factors (97). PPARγ has been shown to reduce oxidative stress and block pro-inflammatory polarization of macrophages. PPARγ regulates ROS production by inhibiting Inducible nitric oxide synthase (iNOS) or enhancing the activity of endothelial nitric oxide synthase(eNOS) (98, 99). Upregulation of antioxidant enzymes as described above can alleviate the condition of FK, and peroxisome can participate in the treatment process of FK through its potential to reduce oxidative stress damage (100). The relative research outcome offers the potential targets for therapeutic strategies to FK.

#### 5.5.3 Inhibition effect of PPARs on inflammations

During the course of FK disease, the retinoid X receptor (RXR) recognizes PAMP and activates a large number of upregulated cytokines/chemokines through signal transduction molecules. Fungal antigens activate CD4+T, activate Th_1_, Th_2_, and Th_17_, and regulate T cell production. The production of related cytokines determines the final outcome of fungal inflammation (101). Chemokines recruit neutrophils, regulate vascularization, play a key role in inflammatory responses, and are widely involved in a variety of complex physiological processes (102, 103). Chemokines are involved in FK pathogenesis mentioned above. CC chemokine receptor 1 (CCR1) is a member of the chemokine family and its receptor family, playing a role in the autoimmune response. Inhibiting the MAPK signaling pathway of CCR_1_ and PPAR - γ reduces inflammatory response and can downregulate the expression of iNOS, IL-1 β, COX-2, and MMP13. Chemokine inhibitors reduce the inflammatory response through the PPARγ pathway (104). PPAR agonists have recently been demonstrated promising short-term biochemical responses in patients with primary cholangitis. PPAR-γ plays a central and important role in the inhibition of inflammation (65). Peroxisome can be exploited to develop new anti-inflammatory drugs to treat FK.

#### 5.5.4 Inhibition effect of PPARs on corneal neovascularization

Infection is an important factor in promoting corneal neovascularization, which can cause corneal opacity and lead to decreased vision. And often leads to chronic inflammatory circulation (105). The balance between angiogenic factors and anti-angiogenic factors is an important cause of corneal neovascularization. Anti-Vascular endothelial growth factor(anti-VEGF) drugs are effective in the treatment of neovascular eye diseases such as age-related macular degeneration, diabetes retinopathy, macular edema, neovascular glaucoma, and other neovascular diseases. Keratitis can cause pathological corneal neovascularization, and the inflammatory genes have a time-dependent effect on capillary regression and corneal transparency recovery. When endogenous capillaries disappear, corneal capillaries become thinner, accompanied by the disappearance of inflammatory cells. Anti-VEGF drugs have great potential in treating corneal neovascularization (106). Endogenous capillary regression is characterized by progressive thinning and remodeling of neovascularization in the rat cornea and *in vivo* inflammatory cell regression. VEGF ligand receptor signaling is inhibited during vascular regression and PPARα/RXRα is activated in the later stages, which may be responsible for inhibiting pro-inflammatory genes and immature blood vessels, and helping to prune and remodel capillaries. The administration of PPARα, a γ agonist, promotes corneal wound healing and provides a new therapeutic strategy (78, 107).

#### 5.5.5 Inhibition effect of PPARs on corneal fibrosis and scar formation

After the corneal injury, transforming growth factor beta-1,2(TGFβ-1,2) is activated and enters the corneal stroma, promoting the transition of keratinocytes near the injury site to myofibroblasts, excreting a large amount of disordered extracellular matrix, forming corneal scarring, and causing vision loss (108–111). PPARγ can inhibit inflammation, fibrosis, and cell differentiation activated by TGFβ (112). Impaired PPARα signaling can affect FXR activation by inducing the TGFβ signaling pathway, P53 signaling pathway, and PI3K-AKT-mTOR signaling pathway, reducing the liver’s ability to inhibit inflammation, and ultimately leading to apoptosis and fibrosis (113–115). PPAR plays an important role in inflammation, angiogenesis, and fibrosis formation, and these pathological processes are also important causes of FK (116). PPAR agonists were considered promising therapeutic targets to reduce corneal neovascularization, fibrosis, and inflammation (117).

#### 5.5.6 Inhibition effect of PPARs on corneal transplant rejection in FK

**Figure 2:**
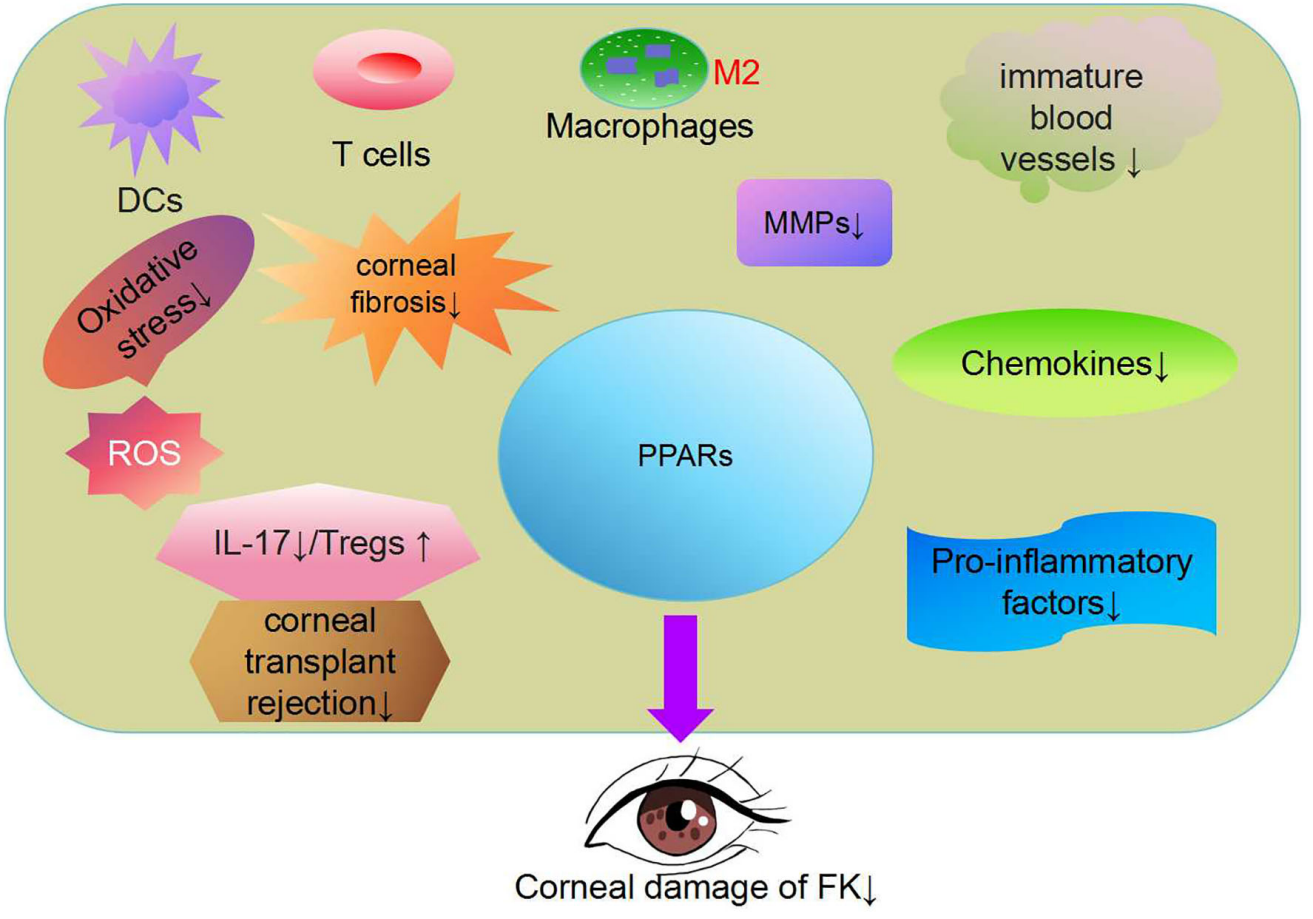
Peroxisome proliferator activated receptor(PPAR) regulates lipid metabolism, antioxidant responses, inflammation and immune factors (97). PPAR-γ has been shown to reduce oxidative stress and block pro-inflammatory polarization in macrophages (78, 108). The PPARα signaling pathway has therapeutic effects in addressing inflammatory corneal angiogenesis and corneal fibrosis (106, 116). PPARγ agonists can significantly proliferate interleukin 17(+) T cells and promote the proliferation of Regulatory T cells (Tregs). PPAR has great potential in the treatment of FK.

In cases of FK, corneal transplantation is an effective measure for restoring vision, but fungal recurrence and corneal transplant rejection are important reasons for surgical failure. In cases of corneal vascularization or severe infection, the failure rate of corneal transplantation can reach as high as 35% to 70% in patients undergoing corneal transplantation. The rejection reaction of corneal transplantation leads to irreversible damage to the corneal endothelium and corneal opacity (118). The loss of donor corneal endothelial cells is progressive after corneal transplant, which can lead to late-stage graft failure (119). T cell activation is an important cause of corneal transplant rejection. Activated T cells proliferate to form CD4+T helper cells, causing a delayed hypersensitive immune response against the same antigen on the transplant, resulting in damage to corneal tissue (120, 121). After T cells recognize foreign antigens, antigen-presenting cells (APCs) trigger innate and adaptive immunity. CD4+T cells differentiate into Th_1_, Th_2_,Treg, and Th_17_ cells, causing tissue inflammation and immune damage (122, 123). PPARγ agonists can significantly inhibit the proliferation of IL-17 (+) T cells and promote the proliferation of Tregs in a heart transplant model, which can help protect heart allografts. Given the anti-inflammatory and anti-immunity effects of PPARγ agonists, the agonists were used to treat acute and chronic allograft rejection reactions (124–126) (**Figure 2**).

## 6 Conclusion

FK is an infectious disease that seriously affects eye health. Due to the widespread use of antibiotics and steroid drugs, dysbiosis of the microbiota leads to a continuous increase in fungal infections. At present, there is a lack of effective antifungal drugs, and fungal resistance remains an urgent problem to be solved. PPAR is a member of the nuclear receptor family, widely expressed in the cornea, and has made research progress in various eye diseases. Excessive immune inflammatory damage in FK cases resulted in severe damage to corneal tissue. The occurrence of corneal transplant rejection is an important reason for the failure of FK vision restoration. Corneal scarring in the late stage of PK is also an important treatment direction. The anti-inflammatory, immunomodulatory, immunosuppressive, antioxidant, and anti-fibrotic functions of PPAR are a promising choice for future FK treatment. This will contribute to better treatment outcomes for patients with FK.
